# GAMETES: a fast, direct algorithm for generating pure, strict, epistatic models with random architectures

**DOI:** 10.1186/1756-0381-5-16

**Published:** 2012-10-01

**Authors:** Ryan J Urbanowicz, Jeff Kiralis, Nicholas A Sinnott-Armstrong, Tamra Heberling, Jonathan M Fisher, Jason H Moore

**Affiliations:** 1 Department of Genetics, Institute for Quantitative Biomedical Sciences, Dartmouth Medical School, Lebanon, NH, USA

**Keywords:** GAMETES, SNP, Epistasis, Simulation, Model, Genetics

## Abstract

**Background:**

Geneticists who look beyond single locus disease associations require additional strategies for the detection of complex multi-locus effects. Epistasis, a multi-locus masking effect, presents a particular challenge, and has been the target of bioinformatic development. Thorough evaluation of new algorithms calls for simulation studies in which known disease models are sought. To date, the best methods for generating simulated multi-locus epistatic models rely on genetic algorithms. However, such methods are computationally expensive, difficult to adapt to multiple objectives, and unlikely to yield models with a precise form of epistasis which we refer to as pure and strict. Purely and strictly epistatic models constitute the worst-case in terms of detecting disease associations, since such associations may only be observed if all *n*-loci are included in the disease model. This makes them an attractive gold standard for simulation studies considering complex multi-locus effects.

**Results:**

We introduce GAMETES, a user-friendly software package and algorithm which generates complex biallelic single nucleotide polymorphism (SNP) disease models for simulation studies. GAMETES rapidly and precisely generates random, pure, strict *n*-locus models with specified genetic constraints. These constraints include heritability, minor allele frequencies of the SNPs, and population prevalence. GAMETES also includes a simple dataset simulation strategy which may be utilized to rapidly generate an archive of simulated datasets for given genetic models. We highlight the utility and limitations of GAMETES with an example simulation study using MDR, an algorithm designed to detect epistasis.

**Conclusions:**

GAMETES is a fast, flexible, and precise tool for generating complex *n*-locus models with random architectures. While GAMETES has a limited ability to generate models with higher heritabilities, it is proficient at generating the lower heritability models typically used in simulation studies evaluating new algorithms. In addition, the GAMETES modeling strategy may be flexibly combined with any dataset simulation strategy. Beyond dataset simulation, GAMETES could be employed to pursue theoretical characterization of genetic models and epistasis.

## Background

Despite the rising quality and abundance of genetic data, epidemiologists continue to struggle to explain the known heritability of these complex phenotypes with existing genetic factors. While strategies seeking single locus associations (i.e. main effects) are often sufficient to address diseases which follow Mendelian patterns of inheritance, their application to diseases characterized as complex has yielded limited success in the pursuit of explaining heritability [1,2]. Epistasis is one of several phenomena reviewed by [3] believed to hinder the reliable identification of predictive genetic markers in association studies. The term *epistasis* was coined to describe a genetic “masking” effect viewed as a multi-locus extension of the dominance phenomenon, where a variant at one locus prevents the variant at another locus from manifesting its effect [4]. In the present study we consider statistical epistasis, which is the phenomenon as it would be observed in association studies. Statistical epistasis is traditionally defined as a deviation from additivity in a mathematical model summarizing the relationship between multi-locus genotypes and phenotypic variation in a population [5]. Specifically, we focus on statistical epistasis that is both *strict* and *pure*. An *n*-locus model is purely and strictly epistatic if all *n* loci, but no fewer, are predictive of disease status. The loci in these models could be viewed as “fully masked” in that no predictive information is gained until all *n* loci are considered in concert.

Computational geneticists are putting significant effort into developing algorithms for the detection of complex disease associations within clinical data [6-9]. While real biological datasets serve as the gold standard for validating new techniques, the development and systematic evaluation of computational strategies calls for simulated data.

Previous genetic data simulation efforts have introduced strategies for generating different data types including: quantitative trait loci mapping [10], pedigree association [11-15], and case/control-based association [16-23]. The simulation of datasets typically involves two stages: model generation, and sample generation. While most of the strategies cited above focus on the latter stage, the generation of realistic complex disease models constitutes a key challenge.

Moore, et. al [18] noted that simple, biallelic, 1-locus or purely epistatic 2-locus models of disease can be assembled with relative ease via trial and error. [24] characterized all fully penetrant two-locus models, where genotype disease probabilities were restricted to zero and one [25], later expanded upon this to include models with continuous penetrance values [26], generated purely epistatic models using the double description method [27] (for 2-locus models) and a non-linear maximization strategy (for 3 and 4-locus models) [17] and [18], recruited an evolutionary algorithm (EA) to evolve 2 to 5-locus epistatic models encoded as binary chromosomes. Over successive generations, models evolved towards a state of pure epistasis. Most recently, [23] circumvented the typical first stage of data simulation (i.e. model generation), directly evolving the genotypes and affection status of samples in a dataset. Evolving datasets with 3 to 5 SNPs at a time, this EA strategy evolved 3 to 5-locus epistatic interactions while attempting to avoid main effects and any intermediate *nested* 2-locus interactions.

While EA’s have offered some success in the automated discovery of complex genetic models, there are distinct drawbacks to their use. First, they are computationally expensive, limiting the order and the quantity of models that can be feasibly generated. Second, EA’s are not guaranteed to find epistatic models that are either pure or strict. Finally, if researchers wish to specify other model constraints (e.g. heritability, or prevalence), the EA fitness landscape becomes multi-objective, introducing further challenges and limitations to the evolutionary process.

In this study we introduce a Genetic Architecture Model Emulator for Testing and Evaluating Software (GAMETES). This algorithm provides a direct approach for the simulation of biallelic *n*-locus epistatic models which may be used in conjunction with any sample generation strategy. Specifically, GAMETES generates a precise class of epistatic models that are both pure and strict. Each *n*-locus model is generated deterministically, based on a set of random parameters, a randomly selected direction, and specified values of heritability, minor allele frequencies, and (optionally) population disease prevalence. For valid combinations of these model constraints, GAMETES attempts to generate a population of model architectures. We use the term *architecture* to reference the unique composition of a model (i.e. the penetrance values and arrangement of those values across genotypes). We demonstrate that GAMETES is a fast, reliable, and flexible method for generating complex genetic models of random architecture. We evaluate GAMETES over an example simulation study using MDR [28], an exhaustive combinatorial search algorithm designed to detect epistasis.

## Methods

In this section, we describe (1) relevant background in genetics and modeling, (2) the specific steps for generating *n*-locus epistatic models that are both strict and pure and (3) an example simulation study using GAMETES.

### Genetics and modeling

Single nucleotide polymorphisms (SNPs) are single loci in the DNA sequence where alternate nucleotides (i.e. alleles) are observed between members of a species or between paired chromosomes in an individual. Most characterized SNPs are biallelic, meaning that only two alleles (*A* or *a*) are observed in a population. The genotype of a SNP in a diploid organism is determined by alleles found on each chromosome of the homologous pair. A biallelic SNP can have one of three genotypes: *AA*, *Aa*, or *aa*. The term *genotype* has been used to refer both to the allele states of a single SNP, as well as the combined allele states of multiple SNPs. To avoid confusion, we refer to the latter as a multi-locus genotype (MLG) whenever necessary.

SNPs not under selective pressure within a population typically exhibit genotype frequencies that are predicted by the Hardy-Weinberg Law [29]. Like most data simulation strategies, GAMETES adopts the assumption of Hardy-Weinberg Equilibrium (HWE) such that the allele frequencies of a SNP may be used to calculate it’s genotype frequencies as follows: freq(*AA*) =*p*^2^, freq(*Aa*) =2*pq*, and freq(*aa*) =*q*^2^, where *p* is the frequency of the major (more common) allele ‘*A*’, *q* is the minor allele frequency (MAF) of the minor allele ‘*a*’, and *p* + *q*=1. GAMETES also assumes that alleles at different loci are in linkage equilibrium.

Penetrance functions, also referred to as penetrance tables, represent one approach to modeling the relationship between genetic variation and risk of disease. Penetrance is the probability of disease, given a particular genotype or MLG. Table 1 gives a penetrance function for a simple autosomal recessive disease model (i.e. a disease that requires two copies of the same allele).

**Table 1:** Penetrance function providing penetrance values for three genotypes from a SNP acting under an autosomal recessive disease model

SNP 1		
AA(.25)	Aa (.5)	aa(.25)
0	0	1

Genotype frequencies are given in parenthesis and *q*=*p*=.5.

This penetrance function is *fully penetrant*, since disease status is completely dependent on genotype (i.e. penetrance values are either 0 or 1). For single-locus models such as this, one can quickly determine if the SNP displays any main effect. The absence of a main effect would manifest itself as equal penetrance values for all three genotypes. Penetrance functions are easily extended to describe *n*-locus interactions between *n* predictive loci using a penetrance function comprised of 3^*n*^ penetrance values corresponding to each of the 3^*n*^ MLGs.

#### Statistical models of epistasis

**Figure 1:**
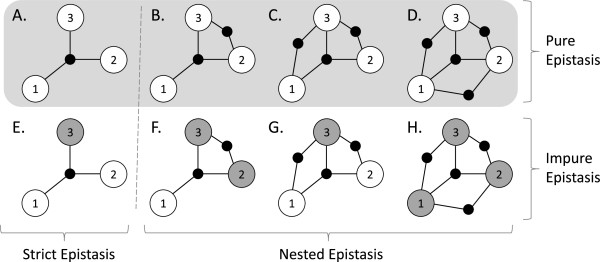
**Illustrations (A) – (H) indicate various types of 3-locus epistatic interactions.** Each numbered circle represents a SNP (where white circles indicate a SNP with no main effect, and darkened circles represent main effects). Each solid black node, with incident edges connecting respective SNPs, represents a 2 or 3-locus epistatic interaction. Illustrations **(A)** – **(D)** exemplify pure epistasis, while **(E)** – **(H)** exemplify impure epistasis. Illustrations **(A)** and **(E)** exemplify strict epistasis, while **(B)** – **(D)** and **(F)** – **(H)** exemplify nested epistasis.

We classify models of statistical epistasis into *pure* and *impure*, as well as *strict* and *nested* subtypes. For each, we say that *n* loci interact epistatically if the heritability of the *n* loci together exceeds the sum of the heritabilities of each of the *n* loci individually. *Pure* refers to epistasis between *n* loci that do not display any main effects [8,26,30]. Alternatively, impure epistasis implies that one or more of the interacting loci have a main effect contributing to disease status (see §1.1 in Additional file 1). *Impure* epistasis should be easier for non-exhaustive data analysis strategies to detect since any main effect(s) could serve as a guide towards the correct multi-locus interaction. *Strict* refers to epistasis where *n* loci are predictive of phenotype but no proper multi-locus subset of them are. By definition, all 2-locus epistatic interactions are strict. *Nested* refers to epistasis in which at least one proper subset of the loci also interact epistatically. Nested epistasis should be easier for non-exhaustive data analysis strategies to detect since the nested subset of loci could serve as a guide towards the correct *n*-locus interaction. To the best of our knowledge, the literature has yet to consider the distinction between strict and nested epistasis. Figure 1 illustrates the distinction between *pure / impure* and *strict / nested*. The present study explores the generation of worst-case models of epistasis, that are both pure and strict. However, the methods we present can be easily extended to the other types of epistatic models just described.

Tables 2 and 3 give examples of epistatic models that are both pure and strict. While fully penetrant models, like those found in Tables 1 and 2 are easy to interpret, they are rarely representative of real interactions. More realistic models, like the one in Table 3 and the ones typically generated by GAMETES, possess continuous penetrance values between 0 and 1. The models from Tables 2 and 3 include a penetrance function relating two SNPs to risk of disease. Each of the nine entries in these tables correspond to one of the nine possible MLGs combining SNPs 1 and 2. For instance, according to Table 2, subjects that have the MLG (*AA-Bb*) are certain to have disease and according to Table 3 subjects that have the MLG (*aa-bb*) have a 14.7*%* chance of having disease. What makes these penetrance functions purely epistatic is that while the genotypes of SNPs 1 and 2 are together predictive of disease status, neither is individually. Indeed, in the case of Table 3, if an individual has genotype AA and SNP 2’s genotype is ignored, the probability he has disease is

(1) $$\begin{array}{lll} .25\cdot .266+.5\cdot .764+.25\cdot .664= & \; & \;\\ \langle .25,.5,.25\rangle \cdot \langle .266,.764,.664\rangle = & .614\; \end{array}$$

which is the marginal penetrance associated with genotype AA. (Computations are to three decimals places.) If he has genotype Aa, the probability of disease is

(2) 〈.25,.5,.25〉·〈.928,.398,.733〉=.614

and for genotype aa it’s

(3) 〈.25,.5,.25〉·〈.456,.927,.147〉=.614.

**Table 2:** A fully penetrant 2-locus purely epistatic penetrance function

		SNP 2			Marginal
	Genotype	BB(.25)	Bb (.5)	bb(.25)	penetrance
	AA(.25)	0	1	0	.5
SNP 1	Aa (.5)	1	0	1	.5
	aa(.25)	0	1	0	.5
	Marginal	.5	.5	.5	K = .5
	Penetrance				

**Table 3:** A 2-locus purely epistatic penetrance function

		SNP 2			Marginal
	Genotype	BB(.25)	Bb (.5)	bb(.25)	penetrance
	AA(.36)	.266	.764	.664	.614
SNP 1	Aa (.48)	.928	.398	.733	.614
	aa(.16)	.456	.927	.147	.614
	Marginal	.614	.614	.614	K = .614
	Penetrance				

Thus, SNP 1’s genotype alone is not predictive of disease status. Similar computations (using the probabilities of SNP 1’s genotypes and the columns in Table 3) give the same value of .614 for the three marginal penetrances associated with SNP 2. Therefore, SNP 2 alone is also not predictive of disease status. The equality of all six of these marginal penetrances is the mathematical definition of strict, pure epistasis for 2-locus models. Their common value equals the population prevalence of disease (K). An expanded definition for *n*-locus models is given in Additional file 1 (§2.3). It essentially says that an (n)-locus model is strictly and purely epistastic if all of the *n* 3^*n*−1^ dot products, analogous to the six just discussed, are equal.

### The GAMETES algorithm

We first outline some of the main ideas GAMETES uses to generate random, pure, strict epistatic models. More detail is given in the following sections, and still more is in Additional file 1 (§2). A key part of the GAMETES algorithm is that certain choices of 2^*n*^ entries of an *n*-way strict, pure epistatic penetrance table uniquely determine the rest of the table. Here we are assuming that the population prevalence is specified. For example, the four entries .764, .398, .733 and .456 of the penetrance table in Table 3 determine the remaining five entries via equations (1), (2) and (3), and the analogous equations involving the columns of the table. So a first attempt to generate random penetrance tables might be to randomly vary these entries or even to randomly seed the four positions in the penetrance table they occupy. Two difficulties arise with this. One is that the mere choice of these positions—the ones that would be randomly seeded—biases the resulting penetrance tables (to have, for instance, high heritabilites). The other is that varying the values, even slightly, of the four chosen entries might not produce a penetrance table. For instance, if the entry .398 of the four chosen ones is changed to .37, then the entry just below it is forced to be greater than one.

This latter difficulty is addressed by working with what we call pre-penetrance tables. These are easily converted to strict, pure, epistatic penetrance tables and are essentially such tables with all marginal penetrances equal to zero. Pre-penetrance tables were used in a somewhat different context from ours in §8 of [5]. There, they arise as the difference between the 2-locus genotypic means of a quantitative trait and the linear model which best fits these means, and so show the wealth of epistatic models. They are our starting point for generating these models, primarily because *all* pre-penetrance tables, unlike strict, pure, epistatic penetrance tables, are easily described: each point of ${\mathbb{R}}^{2^{n}}\;$, Euclidean space of dimension 2^*n*^, corresponds to a unique *n*-locus pre-penetrance table. So picking a random point in ${\mathbb{R}}^{2^{n}}$ (only a random direction is needed) determines a random pre-penetrance table. Converting this to a penetrance table gives our notion of a random, strict, pure, epistatic penetrance table.

There is a catch here which gets back to the first difficulty: the one-to-one correspondence between points of ${\mathbb{R}}^{2^{n}}$ and all pre-penetrance tables depends on the choice of the 2^*n*^ positions that are randomly seeded. The GAMETES algorithm accounts for this by choosing these positions as randomly as is computationally feasible, as discussed in the next section.

**Figure 2:**
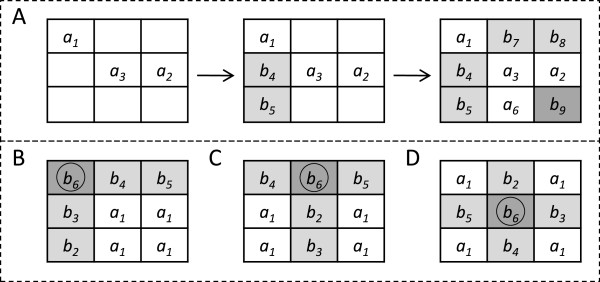
**Examples of our methods for generating parameters for 2-locus pre-penetrance functions.** Example **A**, illustrates the Sudoku method and examples **B**, **C**, and **D** illustrate the Point Method. The *a*’s (white boxes) are independent variables, or parameters, chosen by these two methods. The *b*’s (grey boxes) are dependent variables, specified in terms of the *a*’s, where the dark grey boxes highlight the last variables to be specified. The subscripts indicate one of many orders in which these dependences can be found. In A, the variables *a*_1_, *a*_2_ and *a*_3_ are first chosen by the Sudoku method, then *b*_4_ and *b*_5_ are found in terms of these. After this, one of the four possibilities for *a*_6_ is selected. The remaining *b*’s are then found. In B, C, and D, the circled *b*’s represent example reference points for the Point Method.

We think of a variable as occupying each of these 2^*n*^ positions—the ones which, when seeded, determine all the entries of a pre-penetrance table. We call these 2^*n*^ variables *parameters*. Figure 2 (A,B,C,D) gives four sets of parameter choices out of the 81 that are possible for 2-locus tables. Parameters are independent variables for determining pre-penetrance tables since all entries can be expressed in terms of them.

#### Generating random parameters

A truly random way to pick parameters for *n*-locus pre-penetrance tables is to sequentially pick 2^*n*^ positions at random and then check to see if these give parameters. The check, however, is computationally slow. (It requires determining if a 2^*n*^×2^*n*^ matrix is invertible.) Moreover, many such choices do not give parameters. GAMETES uses what we call the Sudoku method, a much more efficient approach to picking parameters, with only a slight loss of randomness (see Figure 2A). The Sudoku method also picks parameters sequentially. However, after each choice, it expresses as many entries as possible in terms of the chosen parameters, and these are then omitted as possible future choices. This greatly reduces the number of choices which do *not* lead to parameters. Another advantage of the Sudoku method is that if after 2^*n*^ choices all 3^*n*^ entries of an *n*-locus table have been expressed in terms of the chosen ones, then no check is required: the 2^*n*^ choices always give parameters in this case.

The Sudoku method always produces parameters randomly when the number of loci *n* is 2. As *n* grows, two things happen: (1) some bias occurs in parameter generation, and (2) the success rate for finding parameters decreases: for *n*=2,…,8, these rates were 100%, 99.9%, 99%, 92.9%, 61.2%, 3.28%, and 0% based on 750,000 GAMETES runs. Because of this, GAMETES uses what we call the Point method to generate parameters when *n*≥6. Figure 2 (B,C,D) illustrates three iterations of the Point method in the case of two loci. For *n* loci, the method starts by randomly choosing any one of the 3^*n*^ entries. The parameters the Point method then produces are the ones given by the 2^*n*^ entries whose corresponding genotypes differ at every single SNP from the genotype corresponding to the chosen entry. The Point method always succeeds, and since it picks randomly from 3^*n*^ choices of parameters, not much randomness is lost.

#### From parameters to penetrance functions

Any pre-penetrance function can be converted to a pure, strict, epistatic penetrance function by applying a linear scaling function *S* to each of its entries. This function *S* is defined by: the *ij*^th^ entry of *S*(*G*), *G* being any pre-penetrance function, is $S{\left(G\right)}_{\mathrm{ij}}=\frac{g_{\mathrm{ij}}-m}{M-m}$. Here *g*_*ij*_ is the *ij*^th^ entry of *G*, and *M* and *m* are the maximum and minimum respectively, of the entries of *G*. The entries of *S*(*G*) lie in the interval [0,1], with the minimum entry 0 and the maximum 1. Note that if all entries of *G* are multiplied by any positive constant *c*, giving the pre-penetrance table *cG*, then *S*(*cG*)=*S*(*G*). So applying the function *S* to all pre-penetrance tables in the direction of *G*, meaning all positive multiples of *G*, gives the same penetrance table.

Now, given a random direction of pre-penetrance tables, the function *S* converts it to our notion of a random, strict, pure, epistatic penetrance table. Choosing such a random direction requires an explicit description of all pre-penetrance tables. Any parameters, for instance those supplied by one of the methods discussed above, provide such a description. It takes the form of a one-to-one correspondence between all points of ${\mathbb{R}}^{2^{n}}\;$ and all *n*-locus pre-penetrance tables. Specifically, given a point in ${\mathbb{R}}^{2^{n}}\;$, the corresponding pre-penetrance table has parameter values equal to the coordinates of the point. So, given randomly chosen parameters, picking a random direction of *n*-locus pre-penetrance tables now amounts to picking a random direction or, equivalently, a unit vector in ${\mathbb{R}}^{2^{n}}$. We do this using G.W. Brown’s algorithm discussed on page 135 of [31].

#### Adjusting heritability and prevalence

Assume now that a random, pure, strict epistatic penetrance table with a specified heritability, and perhaps also a specified prevalence is desired. Then GAMETES generates a random penetrance table as above and linearly scales its entries to achieve, if possible, the specified values. Linearly scaling the entries, meaning applying a function of the form *f*(*x*)=*mx* + *b*,*m*>0 to each, changes the penetrance table in a relatively minor way so that randomness is preserved. If just heritability is specified, this scaling is done without changing prevalence. The required values of *m* and *b* are discussed in Additional file 1 (§2.5).

Certain values of heritability and prevalence can never be achieved as discussed in the next section. So GAMETES will always fail if these values are specified. Also, penetrance tables with certain other values of heritability and prevalence are very sparse among all penetrance tables and so are generated by GAMETES with extremely low probability. For example, there exist *n*-locus penetrance tables with all heritabilities ≤1 having prevalence and all MAFs equal to $\frac{1}{2}$. However, in both the 5 and 6-locus case, GAMETES generated none of these with heritability ≥.1 after 100,000 iterations.

#### Limits on heritability and prevalence

The values of heritability and prevalence which a penetrance table can have are limited. For instance, no penetrance table has heritability 1 and prevalence $\frac{1}{4}$. The limits are more severe if MAFs are specified. For example, there are 2-locus penetrance tables with heritability 1 and prevalence $\frac{1}{2}$, but none if both MAFs are $\frac{1}{4}$. Penetrance tables with heritability 1 only exist if the MAFs are $\frac{1}{2}$ or $1-\frac{1}{\sqrt{2}}$. See Table 2 for an example of a penetrance table with a heritability of 1.

**Figure 3:**
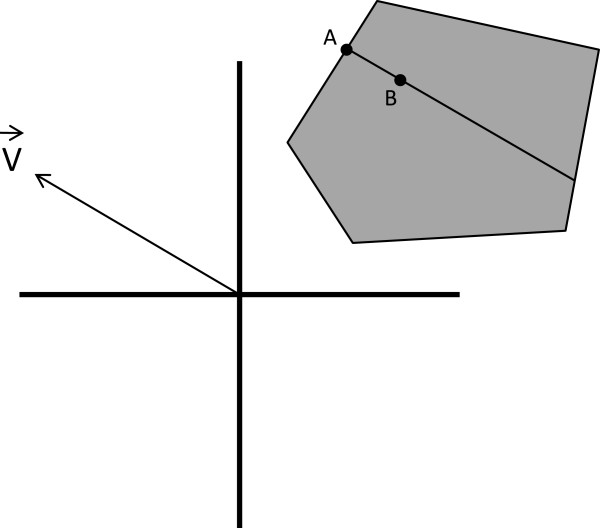
Plot of our maximum heritability estimates for pure, strict, 2-locus models.

Culverhouse, et. al [26] summarized estimates of maximum heritabilities for 2 to 4-locus purely epistatic models over a range of MAFs and prevalence values. Figure 3 illustrates our maximum heritability estimates for pure, strict 2-locus epistatic models where both SNPs have the same MAF. Our method for estimating maximum heritability is discussed in Additional file 1 (§2.6). Generally speaking, if the prevalence *K* is near .5, maximum heritabilities are largest when the MAFs are either 0.5 or $1-\frac{1}{\sqrt{2}}(\approx .29)$, and fall off as the MAFs move away from these values. For other values of *K*, maximum heritabilities tend to increase with MAFs.

#### Summary of the GAMETES Algorithm

The GAMETES algorithm first (1) generates random parameters and a random unit vector in ${\mathbb{R}}^{2^{n}}\;$, then (2) generates a random pre-penetrance table by seeding these parameters using the unit vector, and then (3) uses the function *S* to scale the entries of this random pre-penetrance table to generate a random penetrance table. In case a random penetrance table having a specified heritability, or heritability and prevalence is desired, it further (4) scales the entries of this penetrance table to achieve, if possible, these values. If the Sudoku method fails in step (1) or the scaling in step (4), the algorithm iterates until it either succeeds or a specified iteration limit is reached.

**Figure 4:**
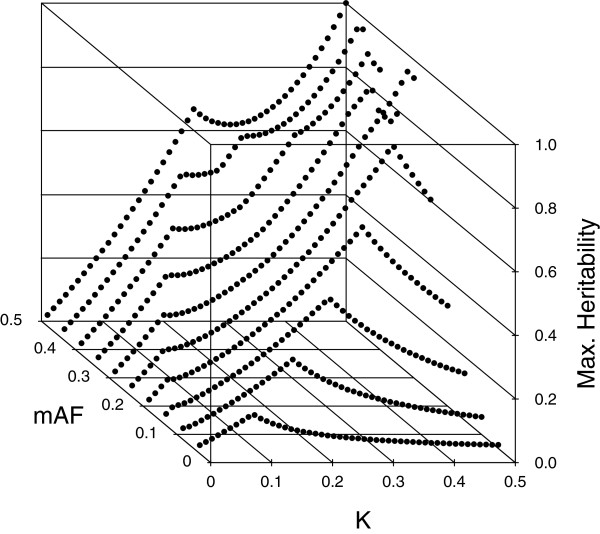
**A schematic depiction of our method for picking random, strict, pure, epistatic penetrance functions.** Points in the parameter space, ${\mathbb{R}}^{2^{n}}$ correspond to pre-penetrance functions, and points in the shaded region correspond to strict, pure, epistatic penetrance functions. The vector $\vec{V}$ indicates a random direction in the set of pre-penetrance functions. $\vec{V}$ determines a random class of penetrance functions, which is indicated by the line in the shaded region. The point *A* on the boundary of this region indicates one (of the two) penetrance functions in this class with maximum heritability. The point *B* indicates the penetrance function in this class having specified values of prevalence and heritability (if one exists). It may be viewed as random among all those with these values.

We give alternative descriptions, illustrated in Figure 4, of steps (2), (3) and (4) to clarify our notion of a random penetrance table. The parameters and vector given by successful completion of step (1) together determine a random direction of pre-penetrance tables. It consists of all pre-penetrance tables obtained by seeding the parameters with points in the direction of the vector. This random direction of pre-penetrance tables determines, in turn, a random class of strict, pure, epistatic penetrance tables. The members of this class consist of all penetrance functions which can be obtained from some pre-penetrance function in the random direction by linearly scaling its entries. These classes, one for each direction of pre-penetrance tables, partition the set of all strict, pure, epistatic penetrance tables. Each (successful) iteration of the GAMETES algorithm essentially picks, at random, one of these classes. The penetrance table in this class with maximum heritability is our random penetrance table. In case a random, strict, pure epistatic penetrance table with specified values of heritability and prevalence is desired, GAMETES picks the unique (if any) penetrance table from this class with these values.

### A GAMETES simulation study

Our evaluation of GAMETES included an assessment of run time, tracking of model generation success, and an example application of GAMETES-simulated datasets to a simulation study using Multifactor Dimensionality Reduction (MDR). MDR is a well documented combinatorial genetics algorithm that exhaustively searches for epistatic interactions [28]. MDR finds models by scanning through all possible combinations of factors up to a pre-specified order of interaction, and has the ability to identify pure, strict epistatic models. MDR was run on a spectrum of simulated datasets, generated using GAMETES. While model generation is the focus of GAMETES, we have included a simple sample generation strategy within the software (see Additional file 1, §2.7). We used GAMETES to attempt generating 5 random models for each of 48 different constraint combinations which differ by number of loci (2, 3, 4, or 5), heritability (0.005, 0.01, 0.025, 0.05, 0.1, or 0.2), and MAF (0.2 or 0.4) with prevalence allowed to vary. GAMETES successfully generated 5 random models for 40 of these constraint combinations. For each of these models, we simulated 100 replicate datasets having balanced sample sizes of 200,400,800, and 1600 (400 datasets per model). For simplicity, each dataset was generated with a total of 20 SNPs. On whole we generated a total of 200 models and 80,000 datasets, all of which were evaluated using MDR.

For every evaluation, MDR was directed to search up to one order higher than the order of the simulated model. The best model was selected based on cross validation (CV) consistency, and in the event of a CV tie, based on testing accuracy. In this study, model detection success was evaluated, referring to the proportion of datasets within which the correct underlying model was identified. Detection success was considered to be meaningful when it was greater than 0.8.

## Results and discussion

Table 4 gives the average run time required for GAMETES to make 100, 000 *n*-locus model generation attempts. Each average is based on 8 different runs of GAMETES in which pure, strict, epistatic models were sought with heritabilities of 0.005, 0.01, 0.025, 0.05, 0.1, 0.2, 0.3, or 0.4 (MAFs =0.5 and K 0.5). Run times were obtained running GAMETES on a 64-bit Dell PC with a 2.5GHz Intel Xeon Processor. The difference between run times for different heritabilities was negligible.

**Table 4:** Average *n*-locus run time of GAMETES

	*** n*-Loci**	**2**	**3**	**4**	**5**	**6**
	Time(sec.)	2.5	5.5	15.3	46.7	153.2

(100,000 iterations).

In addition to run time, we tracked the number of times a model was successfully generated having the specified set of model constraints. Table 5 indicates the frequency of successful model generation for each of the 8 specified heritabilities over all *n*-locus combinations explored. This frequency gives us an indication of how many iterations it takes to find a pure, strict, epistatic model with these specified heritabilities. As we approach the limits of what constraint combinations can be generated it takes GAMETES more attempts to successfully generate such models. In general, models of higher heritability take more attempts to find. This trend is exaggerated as the number of loci (*n*) is increased. The low likelihood of GAMETES to find models of high heritability is an expected limitation of the algorithm, since random models may only be scaled down to lower heritabilities during the model generation process.

**Table 5:** Model generation frequency

	***n*-Loci**	**2**	**3**	**4**	**5**	**6**
	**Heritability**	**Generation Frequency**				
	0.005	1	.99	.99	.93	.71
	0.01	1	.99	.99	.88	.24
	0.025	1	.99	.85	.16	<.01
	0.05	1	.93	.17	<.01	<.01
	0.1	1	.24	<.01	0	0
	0.2	.33	.01	0	0	0
	0.3	.10	<.01	0	0	0
	0.4	.02	<.01	0	0	0

(based on 100,000 attempts).

In Additional file 1 (§3) we give the results of an example simulation study evaluating MDR using the models and datasets generated with GAMETES as previously described. We find that GAMETES is able to generate models useful for such evaluations, i.e. models with constraints at the boundary of what MDR is able todetect.

## Conclusions

This study introduces GAMETES, a fast and reliable strategy for the generation of complex genetic models with random architectures. Specifically, we focus on the generation of pure, strict n-locus epistatic models (considered to be the most difficult to detect). The benefits of our strategy include (1) speed; deterministic calculation of models makes our approach much faster than an EA approach, (2) randomness; models are generated using a strategy which seeks to maximize the randomness of model architecture, (3) the ability to precisely specify genetic constraints, (4) the ability to generate a large population of models from which to choose, and (5) the potential to combine the GAMETES modeling strategy with any data simulation strategy. An obvious limitation of this approach is the difficulty it has finding models of higher heritability. However, GAMETES is proficient at finding models with heritabilities typically used in evaluating and comparing new bioinformatic strategies.

While the probability is low that these types of ‘extreme’ epistatic interactions occur in biology by chance alone, we instead focus on the fact that they ‘can’ occur. With that in mind, our focus on pure strict epistasis is intended to promote the development of strategies that can accommodate even the most challenging relationships. In doing so, we make minimal assumptions about the true nature of biological interaction. Notably, the GAMETES strategy may be extended to produce impure epistatic models, as well as nested epistasic models, which are more likely to occur by chance. This extension will be a focus of future work.

The GAMETES software is open source and freely available for download. It offers an intuitive and flexible framework for the simulation of complex genetic models, and the option to generate simulated datasets from these models. This software offers both a graphical user interface, as well as command line accessibility to facilitate the quick generation of a large simulated dataset archive. The GAMETES software along with a detailed users guide is included as Additional files 2 and 3, respectively.

## Availability and requirements

**Project name:** GAMETES

**Project home page:**http://sourceforge.net/projects/gametes/files/

**Operating systems:** Linux, Mac, PC

**Programming languages:** Java

**Other requirements:** None

**License:** None

## Competing interests

The authors declare that they have no competing interests.

## Author’s contributions

RU co-developed the methodology and software, carried out experiments, and co-wrote the manuscript. JK devolped the mathematical proofs, co-developed the methodology, and co-wrote the methods and supplemental material of the manuscript. NSA co-developed the computational methodology, and carried out the run-time experiment. TH carried out experiment determining maximum heritabilities and worked on respective figure. JF co-developed the software, and programmed the GAMETES GUI software. JH co-developed software. All authors read and approved the final manuscript.

## Abbreviations

SNP: Single nucleotide polymorphism; EA: Evolutionary Algorithm; GAMETES: Genetic Architecture Model Emulator for Testing and Evaluating Software; MLG: Multi-locus genotype; HWE: Hardy-Weinberg Equilibrium; MAF: Minor Allele Frequency; K: Population Prevalence.
